# Identification of apelin/APJ signaling dysregulation in a human iPSC-derived granulosa cell model of Turner syndrome

**DOI:** 10.1038/s41420-024-02231-9

**Published:** 2024-11-14

**Authors:** Wei-Ju Chen, Yi-Ya Chao, Wei-Kai Huang, Wei-Fang Chang, Chii-Ruey Tzeng, Chi-Hsuan Chuang, Pei-Lun Lai, Scott C. Schuyler, Long-Yuan Li, Jean Lu

**Affiliations:** 1 Genomics Research Center, Academia Sinica, Taipei, 11529 Taiwan; 2 Taipei Fertility Center, Taipei, 110 Taiwan; 3 Taipei Medical Technology Co., Ltd, Taipei, 110 Taiwan; 4 Department of Obstetrics and Gynecology, School of Medicine, College of Medicine, Taipei Medical University, Taipei, 110301 Taiwan; 5 Department of Biomedical Sciences, College of Medicine, Chang Gung University, Division of Head and Neck Surgery, Department of Otolaryngology, Chang Gung Memorial Hospital, Taoyuan, 33302 Taiwan; 6 Department of Life Sciences, National Chung Hsing University, Taichung, 402202 Taiwan; 7 Department of Biomedical Sciences and Engineering, Tzu Chi University, Hualien, 97004 Taiwan; 8 Graduate Institute of Medical Sciences, National Defense Medical Center, Taipei, 11490 Taiwan; 9 Genomics and System Biology Program, College of Life Science, National Taiwan University, Taipei, 10617 Taiwan

**Keywords:** Infertility, Stem-cell differentiation

## Abstract

The interaction between germ cells and somatic cells in the ovaries plays a crucial role in establishing the follicle reserve in mammals. Turner syndrome (TS) predominantly affects females who have a partial or complete loss of one X chromosome. Our understanding of the role that granulosa cells (GCs) play in TS disease progression and pathogenesis remains limited. In this study, we achieved GC differentiation efficiency of up to 80% from iPSCs. When attempting to replicate the differentiation process of embryonic granulosa cells, we observed the downregulation of specific genes—*GATA4*, *FOXL2*, *AMHR2*, *CYP19A1*, and *FSH*—in Turner syndrome-derived granulosa cells (TS-GCs). Additionally, we identified dysregulation of the cell cycle in TS-GCs. To uncover the endogenous defects in TS-GCs, we compared global transcriptome patterns between iPSC-derived granulosa cells from healthy individuals and those with Turner syndrome. The apelin/APJ pathway exhibited differential signaling between the healthy and TS groups. Supplementation with apelin ligands and activation of apelin/APJ downstream signaling via Akt/PKB restored cell cycle progression and marker gene expression. We hypothesize that during early embryonic development, failures in apelin/APJ signaling in GCs of Turner syndrome patients lead to abnormalities in ovarian development, ultimately resulting in early oocyte loss and infertility.

## Introduction

Production of functional oocytes, or eggs, depends on complex communication with the surrounding supporting cells and the oocytes. These supporting cells, granulosa cells (GCs), are responsible for follicle formation and secrete cytokines, such as anti-Müllerian hormone (AMH), activins, and hormones. For example, estrogens help oocytes mature in a paracrine fashion. During mammal development, the pluripotent epiblasts undergo multiple steps to form the embryonic gonads. The initiation of gonad development occurs with the formation of a bipotential genital ridge, which is distinguished by the thickening of the coelomic epithelium. This process relies on multiple transcription factors, including *GATA4* (GATA binding protein 4) [1]. Once the genital ridge is formed, pre-supporting cells in individuals with XX and XY chromosomes differentiate into the granulosa or Sertoli cell lineages, respectively. Meanwhile, the primordial germ cells (PGCs) migrate from the hindgut towards the genital ridges, where they merge with each other and also with somatic cells, resulting in the formation of the primary sex cords between 4 and 6 weeks post-conception (wpc) in the female embryonic gonads. The WNT/β-catenin signaling pathway contributes to cell proliferation by increasing GATA4-expressing supporting cells in the developing ovary [2]. Forkhead box L2 (*FOXL2*) is the key transcription factor for maintaining GC identity throughout ovarian development in females by repressing the Sertoli cell marker *SOX9* expression [3]. FOXL2 also cooperates with BMP2 and WNT4 to activate follistatin (*FST*) expression, which is involved in follicle assembly [4], and *CYP19A1*, which encodes the aromatase, responsible for the last step of estrogen production in GCs [5].

Turner syndrome occurs specifically in the female with complete or partial loss of one X chromosome in somatic (body) cells. There are 1/2500 ~ 1/3000 affected individuals in live births with Turner syndrome and about 1% of these newborns can survive to adulthood. >99% 45,XO fetuses abort, typically by 28 weeks of gestation. Among the survivors, 40%–50% postnatal TS patients are pure “monosomy X” (~80% are maternal X) with a 45,XO karyotype. Monosomy X TS suffers from haploinsufficiency of multiple genes on the X chromosome which causes many severe phenotypes, such as embryologic developmental problems, short stature, and defects in gonadal function. 45,X mosaic TS, for example, 46,X,i(Xq), 46,XX, 47,XXX, 46,X,del(Xp), or 46,XY are also the common karyotypes [6, 7]. Typically an isochromosome of the X chromosome, consisting of two long arms of the X chromosome and designated as 46,X,i(Xq), is the most frequent of the mosaic cell lines that have been investigated [8]. In mosaic TS cases, a ring X chromosome with a karyotype 46,X,r(X) is found in approximately 6% of the patients with TS and these patients often have additional unique clinical phenotypes such as mental disorders and learning disabilities [9]. A study suggested that the severity of clinical manifestations in the mosaic ring-X chromosome in a patient is dependent on the degree of mosaicism and the extent of the Xq deletion [10]. Most TS patients have delayed puberty (60%–90%), and 90% of individuals with the 45,X karyotype are infertile, whereas, individuals with a mosaic karyotype have 30%–58% sexual infantilism and show different degrees of primary amenorrhea [8, 11]. The critical regions link to premature ovarian failure (POF) are Xq13.3 to Xq27, Xq21 to Xqter, and Xq13.3–Xq21.1. X-linked genes in these regions include *XPNPEP2* (Xq25), *POF1B* (Xq21.2), *DACH2* (Xq21.3), *CHM* (Xq21.2), *DIAPH2* (Xq22), and *FMR1* (Xq27). For example, *DACH2* and *POF1B* are susceptibility genes linked to premature ovarian failure [12]. *FMR1* is a critical gene in Fragile X Syndrome and premature ovarian failure [13]. Moreover, the proximal Xp region also plays a key role in ovarian function, such as *ZFX* (Xp22.1–21.3) and *BMP15* (Xp11.2) [14].

Induced pluripotent stem cells (iPSCs) are increasingly used to model human disorders. Developing cell therapies using iPSC-derived cells holds promise as a great tool for cell replacement or drug discovery studies. We report here the culturing of Turner syndrome patient-derived cells for studying disease etiology at the molecular and cellular levels, as well as for developing therapies in the future. The derivation of iPSCs from multiple patients is direct. This method has enabled us to analyze similar mutations in diverse genetic backgrounds. Furthermore, when considering models of genetically complex disorders, which often involve multiple unknown loci, this approach is more beneficial than genome editing using normal hPSCs. Finally, the murine model for Turner syndrome is the XO mouse. However, unlike their human infertile counterparts, XO mice are typically fertile [15, 16], which means mouse XO model cannot be used as a model system to fully understand infertility in human TS patients. Because the 45,XO karyotype in humans displays the highest degree of infertility, we aim to utilize 45, XO patient-derived cell lines to investigate the problem of infertility in TS patients.

Mammalian females are born with a predetermined number of ovarian follicles. During early development, disruptions in the proper communication between germ cells and somatic cells can result in ovarian developmental failure. This defect occurs in the oocyte precursor or somatic supporting cells, meaning it originates within the affected cells themselves, and can lead to impaired oocyte differentiation. It results in the reduction in the ovarian follicle reserve, and ultimately, a premature decline in fertility. To our knowledge, one study on GCs in TS addressed that GCs were largely 45,XO and exhibited varying degrees of X chromosome mosaicism among patients or even within follicles in the same patient [17]. In contrast, oocytes in most TS patients (~91%) with mosaic aneuploidy of sex chromosomes had a normal X chromosomal content. Proper oogenesis is highly dependent on the somatic microenvironment. Evidence has implied that malfunctions in GCs will lead to reproductive disorders and aging of ovaries [18, 19]. GCs encapsulate the primordial germ cells (PGCs) and support egg development and let mitotic PGC enter meiosis, a key step in germ cell development. Here, we hypothesize that TS is a GC-mediated disease and GCs dysfunction contributes to ovarian dysfunction. To this end, we developed and employed an optimized granulosa cell differentiation protocol to exam the granulosa cells’ function as a “disease in a dish” model using iPSCs. We differentiated iPSCs reprogrammed from TS probands and unaffected individual fibroblasts into ovarian granulosa cells. Firstly, in the TS-GCs we observed a longer G1 phase arrest and decreased S phase, decreased percentages of cells positive for both ki67 and BrdU which suggest that TS-GCs have a reduced capacity for cell division and replication. Furthermore, TS-GCs displayed lower levels of key genes involved in granulosa cell function. Specifically, the expression of *CYP19A1*, which is essential for estrogen synthesis, as well as *AMHR2* and *FSHR*, which are receptors important for follicle development and hormone signaling, were decreased in TS-GCs. Moreover, the secretion level of anti-Müllerian hormone (AMH), a hormone produced by granulosa cells, was also not detectable in the culture medium of TS-GCs. These findings contribute to our understanding of the impact of the Turner syndrome karyotype in connection with granulosa cell function and follicular development.

We also observed that the apelin/APJ signaling pathway and cytidine triphosphate synthase 2 (*CTPS2*) were downregulated in Turner syndrome iPSC-derived granulosa cells. We found that blocking the activity of the apelin receptor using ML221 resulted in the downregulation of *CTPS2* expression. Furthermore, by treating TS-GCs with APLN-13, ELA-32 (agonists of the apelin/APJ pathway), and SC-79 (an activator of Akt/PKB phosphorylation), we were able to partially repress the cell division defect and GC marker expression observed in TS-GCs. These findings suggest a potential role of the apelin/APJ pathway in regulating *CTPS2* expression and the cell division process in TS-GCs.

## Results

### The optimization of granulosa cell differentiation directly from human iPSCs

Fibroblasts derived from two TS patients (cell line TS1 and TS2), clinically diagnosed and genetically confirmed for having Turner syndrome based on the data records in the Coriell Institute, were reprogrammed into iPSCs [20]. Two control iPSCs were derived from unrelated, unaffected individuals (cell line WT2 and WT4). Karyotype analysis of the iPSC lines was performed and revealed normal 46,XX and 45,X karyotypes, respectively as shown in Fig. S1 and in our previous study [20]. The transcription factors, OCT4, NANOG, SOX2, and the cell surface markers SSEA3 and SSEA4 associated with pluripotency were evaluated by immunostaining of iPSC colonies [20]. No significant differences were discerned among all the iPSC lines regarding their expression of pluripotency markers [20]. The three germ layers differentiation abilities were also characterized in our previous study [20].

**Fig. 1:**
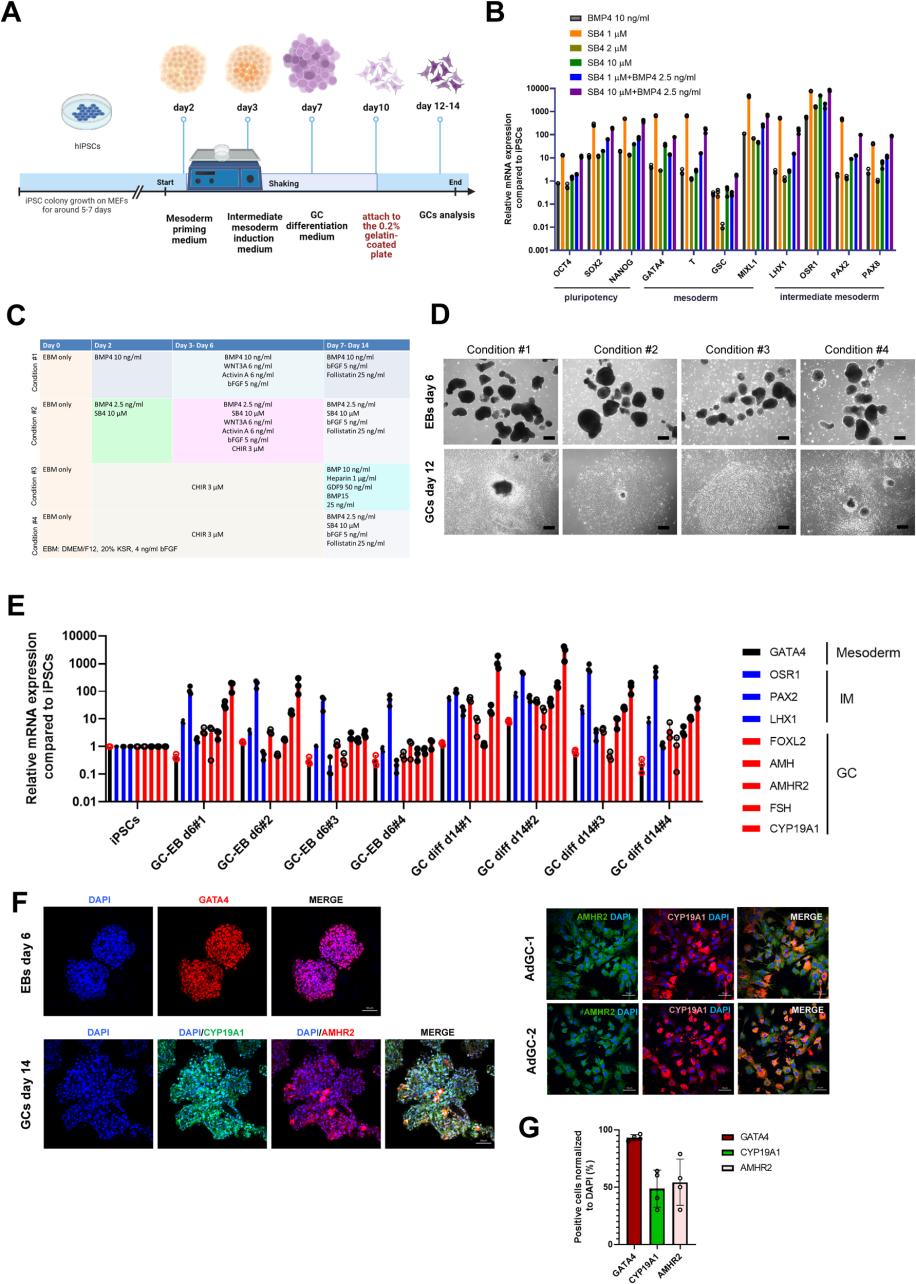
The optimization and characterization of granulosa-like cell differentiation protocols from human induced pluripotent stem cells (iPSCs). **A** Schematic representing of a three-stage approach (mesoderm, intermediate mesoderm (IM), granulosa cells (GCs)) to generate patient-specific models of GC-like cells by guided differentiation of iPSCs. **B** mRNA expression levels of key genes in stem cell pluripotency (differentiation day 0), mesoderm (differentiation day 2), and intermediate mesoderm cells (differentiation day 4–6) by using qRT-PCR analysis under different combinations of SB4 compound and/or BMP4. **C** A representative table depicting small molecular chemicals and recombinant proteins used in four tested conditions of GC differentiation in this study. **D** Brightfield representative images showing the intermediate mesoderm embryonic bodies (EBs) before attachments (day 6) and as differentiated GC (day 12). **E** Comparative examination of mRNA expression levels of mesoderm, IM, GC marker genes between four different differentiation conditions. **F** Immunofluorescence (IF) staining characterizations of mesoderm marker (GATA4) and GC markers (AMHR2, CYP19A1) in differentiation condition #2. The luteinized cumulus granulosa cells served as IF positive control cells. Scale bar, 50 μm. **G** The quantification of fluorescence images of (**F**) (left panel). Bars indicate the mean ± SD (*n* = 4).

Here, we set out to generate a human-derived in vitro granulosa cell model from iPSCs using a modified protocol based on a previously established protocol (condition #1) [21] (Fig. 1A). Briefly, the iPSC colonies were suspended to form embryonic bodies to mimic the embryo development and prime the epiblasts to differentiation into mesoderm cultured in mesoderm patterning medium (Fig. 1A). After that, we further induced the cell fate into intermediate mesoderm, which includes somatic precursors of the gonads, where the middle region from the notochord (medio-lateral axis) during gastrulation was present [22]. BMPs (bone morphogenetic proteins) play a pivotal role in specifying growth regions along the dorsal-ventral axis of the mesoderm and are crucial in the formation of the intermediate mesoderm [23]. In order to induce the profound differentiation direction to intermediate mesoderm, we tried to use SB4, a selective agonist of canonical BMP4 signaling by increasing p-SMAD-1/5/9 activation [24]. We found that using 1 μM of SB4 and 10 μM of SB4 co-modulated with 2.5 ng of BMP4 protein showed higher mesoderm (M) marker expression (e.g., *GATA4*) at day 4 as well as intermediate mesoderm (IM) marker (e.g., *OSR1, PAX2, LHX1*) expression at differentiation day 6 (Fig. 1B). Aside from BMP4 signaling, driving pluripotent stem cell differentiation towards mesendoderm and mesoderm lineages also connects with induction by canonical Wnt and Activin-Nodal signaling [25, 26]. We tested different protocols to induce hiPSCs to mesoderm-like cells by CHIR99021 (condition #3 and condition #4) (Fig. 1C), at differentiation day 7–14, where the cells were guided by the differentiation cues of bone morphogenetic protein 4 (BMP4) signaling and growth signals from germ cells. We found that protocol condition #2 using the SB4 compound co-modulated with BMP4 protein yielded the highest granulosa cell marker expression (e.g., *FOXL2*, *AMH*, *AMHR2*, *FSH*, *CYP19A1*) at differentiation day 14 using qRT-PCR analysis (Fig. 1C–E). However, condition #3 or #4 in which iPSCs were induced by the CHIR99021 method followed by BMP4 signaling did not display high GC marker expression (Fig. 1C–E). Using primary adult GCs obtained from luteinized cumulus granulosa cells during In Vitro Fertilization (IVF) procedures as an immunofluorescence (IF) staining positive control, we also confirmed that the granulosa cell marker, type 2 AMH receptor (AMHR2) and aromatase cytochrome P450 19A1 (CYP19A1), were highly expressed in differentiated granulosa cells at day 14 (Fig. 1F, G).

### TS-GCs recapitulate the ovarian deficit phenotypes in vitro

**Fig. 2:**
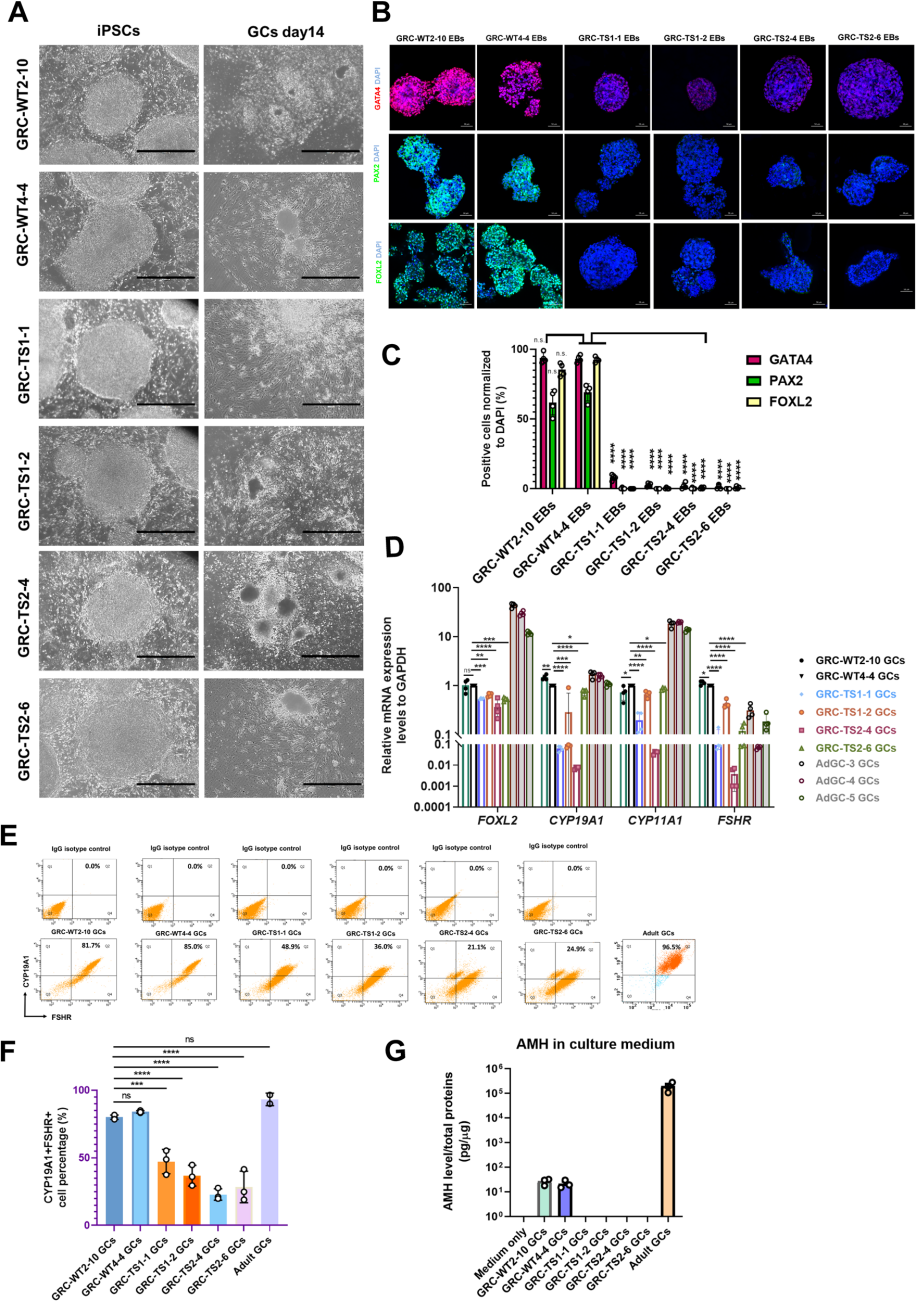
Granulosa cells derived from Turner syndrome patients display differentiation defects and downregulate anti-Müllerian hormone (AMH) secretion capabilities. **A** Representative brightfield images of hiPSCs and the differentiated GCs derived from healthy control (WT) fibroblasts and Turner syndrome (TS) fibroblasts. Scale bar, 1000 μm. The experimental GC differentiation were performed three times independently. **B** Representative immunofluorescence (IF) staining characterizations of GATA4 (mesoderm marker), PAX2 (intermediate mesoderm marker), and FOXL2 (GC-specific marker) in WT-GC and TS-GC clones during each time point of GC differentiation. Scale bar, 50 μm. The experimental GC differentiation were performed at least three times independently. **C** Quantification of fluorescence images of (**B**). Bars indicate the mean ± SD (*n* = 4). ****p < 0.0001 vs. GRC-WT4-4 EBs. **D** Quantitative analysis of the expression of specific genes in GCs differentiated from iPSCs (day 14) and in luteinized cumulus granulosa cells. Bars indicate the mean ± SD (*n* = 4). *p < 0.05, **p < 0.01, ***p < 0.001, and ****p < 0.0001 vs. GRC-WT4-4 GCs. **E** Flow cytometry analysis of GC markers, CYP19A1 and FSHR between healthy control (WT) GCs and TS-GCs. Bars indicate the mean ± SD (*n* = 3). The luteinized cumulus granulosa cells from two adult donors served as staining positive control samples. **F** The flow cytometry quantification result of (**E**). Bars indicate the mean ± SD (*n* = 3 for iPSC-derived GCs; *n* = 2 for adult GCs). ***p < 0.001, and ****p < 0.0001 vs. GRC-WT2-10 GCs. **G** AMH ELISA assays were performed to quantify the AMH levels in the culture supernatants collected from healthy control (WT) GCs and TS-GCs cultured for 3 days. The supernatant collected from the luteinized cumulus granulosa cells served as positive control cells. Secreted AMH levels were normalized to the total protein amounts of the cultured cells. Bars indicate the mean ± SD (*n* = 3).

The hiPSCs-TS from two patients with two subclones (GRC-TS1-1, GRC-TS1-2, GRC-TS2-4, GRC-TS2-6) were compared with two control cell lines (GRC-WT2-10, GRC-WT4-4) for further characterization and analyses. Also, primary adult GCs obtained from luteinized cumulus granulosa cells from non-carrier females were used as a positive control in the characterization experiments. Morphologically, there were no notable differences between TS-GCs and control GCs (Fig. 2A). We found that the expression of GATA4 (mesoderm marker, at differentiation day 4), PAX2 (intermediate mesoderm marker, at differentiation day 6), FOXL2 (GC early marker, at differentiation day 10) were decreased in TS-derived GCs (Fig. 2B, C). These data suggested that TS-iPSCs differentiation potential was slightly lower compared with the healthy control iPSCs during induction of granulosa cell differentiation in vitro. Quantitative real-time PCR (qRT-PCR) analyses of the expression profiles of GC specific markers critical for cell proliferation and differentiation, such as *FOXL2*, revealed that they were downregulated (Fig. 2D). In addition, genes involved in steroidogenesis, for instance, *CYP19A1*, *CYP11A1*, and *FSHR*, were also downregulated in TS-GCs at differentiation day 14 (Fig. 2D). The specific value for the TS-GCs double-positive cell population of FSHR and CYP19A1 were approximately 82.03% in wild-type GC, 41.99% in TS1-GCs, and 25.68% in TS2-GCs (Fig. 2E, F). Anti-Müllerian hormone (AMH) is secreted exclusively in granulosa cells in females and is largely expressed throughout folliculogenesis, from the primary follicular stage towards the antral stage [27]. To test functionality of GCs we differentiated them in vitro, and AMH ELISA tests revealed that the AMH secretion was detected in control iPSC-derived GCs, however, AMH levels were downregulated significantly in cultured medium of both TS1-GCs and TS2-GCs (Fig. 2G), which is reminiscent of observations in XO TS patients having very low or nearly undetectable AMH serum levels [28].

### Transcriptome analysis of iPSC-derived granulosa cells shows TS-derived GCs exhibit aberrant differentiation and altered APLN/APJ signaling

**Fig. 3:**
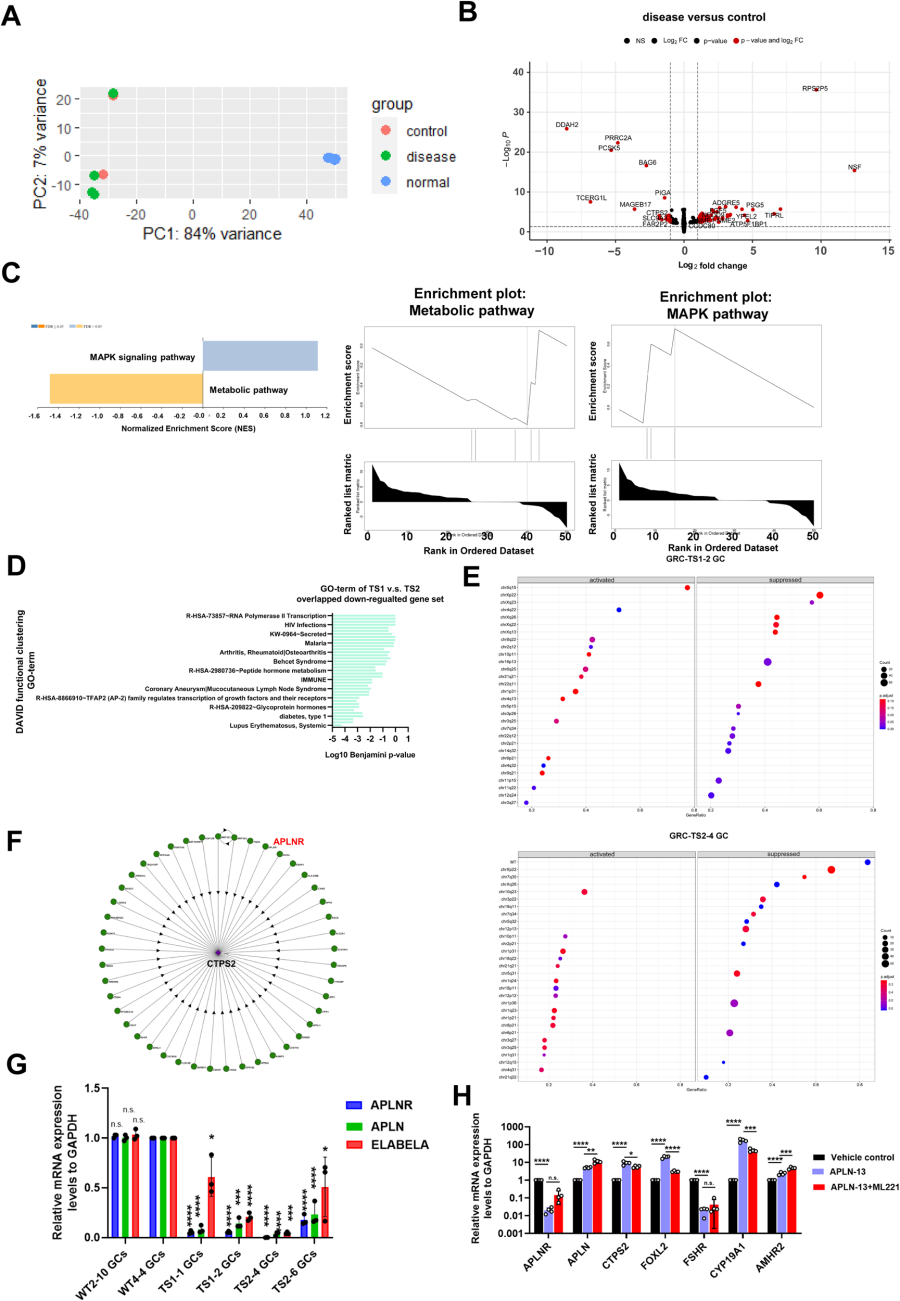
RNA-seq analysis of iPSC-derived GCs reveals disruption of immune associated pathways and metabolic pathways. **A** A principal component analysis (PCA) plot showing separation of the control (WT-GCs: WT2-10, WT4-4), disease (TS-GCs:TS1-1, TS1-2, TS2-4, TS2-6), and normal (luteinized cumulus cells from four doners) GCs samples at differentiation day 12. **B** Volcano plot showing the differentially expressed genes comparing GCs from control and disease samples. Red dots represent genes with |log_2_(fold-change)| >1 and *p*adj < 0.05. The two control iPSC line clones are derived from two unaffected donors, while disease iPSC line clones are derived from two independent patient donors. Two biological replicates are shown for each condition. **C** Gene Set Enrichment Analysis (GSEA) identifying enriched pathways in metabolic pathway with gene set enrichment at the bottom of the ranked list. While a positive normalized enrichment score (NES) indicates gene set enrichment at the top of the ranked list showed MAPK pathway enrichment. NES is the normalized enrichment score to account for the size of each gene set. **D** Bar plot showing enriched gene ontology (GO) terms using DAVID functional annotation analysis for downregulated genes common between GCs derived from TS1 iPSC lines and TS2 iPSC line compared to control lines. **E** GESA analysis based on gene sets in MSigDB databased on C1 category (corresponding to human chromosome cytogenetic bands) in GRC-TS1-2 GCs and GRC-TS2-4 GCs, separately. **F** Illustration of CTSP2 protein interaction map. **G** qRT-PCR analysis to quantified APLNR, APLN, ELABELA expression in control-GCs and TS-GCs. Error bars indicate the mean ± SD (n=3). One-way ANOVA with a Dunnett post hoc test was performed. Multiple comparisons between TS-GCs vs. WT4-4 GCs. *p < 0.05, ***p < 0.001, and ****p < 0.0001. **H** qRT-PCR analysis was performed to determine the mRNA levels of target genes after APLN-13 treatments (20 μM) or with ML221 (10 μM) from differentiation day 5 to day 12. Error bars indicate the mean ± SD (*n* = 4). Unpaired Student’s *t* test was performed. *p < 0.05, **p < 0.01, ***p < 0.001, and ****p < 0.0001.

We conjectured that TS may be, in part, a GC modulated disease, which consequently contributes to loss of oocyte maturation leading to ovarian haploinsufficiency. To investigate potential cellular and molecular deficits in TS-GCs, we performed total RNA-sequencing analyses of granulosa cells directly differentiated from one control cell with two controls (WT2-10 and WT4-4) and two TS patients (TS1 and TS2) with two subclones as biological replicates, respectively. Principal-component and correlation analyses showed clustering of biological replicates of iPSC-derived GCs from four luteinized adult granulosa cells (cumulus GC) which were used as a reference for normal adult GCs (Fig. 3A). The first and second principal component explain 84 and 7 percent of the variance respectively. To investigate potential differences in gene regulation between disease-derived granulosa cells (GCs) and control GCs, we analyzed the differentially expressed genes (DEGs) and identified 10 downregulated genes and 25 upregulated genes at differentiation day 14 (adjusted-*p* value < 0.05; fold change [FC] of log_2_ transformed normalized counts ≧1; Fig. 3B). To gain further understanding of the biological pathways affected in disease-derived granulosa cells (GCs) compared to control GCs, we conducted pathway analysis using gene set enrichment analysis (GSEA) software [29] using all DEGs with an adjusted-*p* value less than 0.05. Although the size number of DEGs was small, we identified metabolic pathways (FDR = 0.062937; leading edge genes: *CTPS2*, *PIGA*) that were enriched in the downregulated gene set. On the other hand, the MAPK signaling pathway (FDR = 0.32996; leading edge genes: *DAXX*, *EGF*, *FGF5*) was enriched in the upregulated gene set (Fig. 3C, D). Using DAVID functional annotation analysis of these overlapping down-regulated DEGs (>1.5 fold change), several key terms, such as RNA polymerase II transcription, immune, and glycoprotein hormone were enriched (Fig. 3D). The pathway enrichment analyses suggested that these biological processes are dysregulated in disease GCs compared to control GCs, highlighting that these pathways changed in disease GCs. We also performed GSEA analyses to compare the gene set and gene sets in the MSigDB database based on C1 (corresponding to human chromosome cytogenetic bands) (Fig. 3E). We observed that the highest downregulated DEGs in TS1-2-GC and TS2-4-GC were correlated to the X chromosome (Fig. 3E). This data corroborates the notion that X chromosome-linked gene defects occur in TS patients.

Since we found that a metabolic pathway was enriched in the downregulated gene set in the DEGs GSEA analyses, we focused on cytidine triphosphate synthase 2 (*CTPS2*) (Fig. 3B, C). The *CTPS2* gene is located in Xp22.2 and is a crucial rate-limiting metabolic enzyme in controlling the synthesis of cytosine nucleotides, which are essential for various metabolic processes and provide the building blocks required for the synthesis of RNA and DNA. CTPS2 was reported as being involved in cell growth, and loss of CTPS2 may lead to cell cycle arrest and apoptosis in chronic lymphocytic leukemia cells [30]. CTPS2 was inferred to interact with APLNR in a mass spectrometry experiment in HCT116 cells [31] (Fig. 3F). *APLN* encodes the apelin protein, a ligand of *APLNR*, and is located on the long arm of the X chromosome at position Xq 25–26. The N-terminus of the protein contains signal sequences involved in the ligand–receptor interaction. The C-terminus plays a crucial role in maintaining the biological activity of the ligand. After post-translational modification, the 77-amino acid pre-pro-peptide encoded by *APLN* is transformed into the endogenous functional isoforms (apelin-12, apelin-13, apelin-17, apelin-36). Research has found shorter chains, such as apelin-13 (APLN-13), are characterized as having higher biological activity. We also confirmed that the downregulated expression of *APLN* and *APLNR* in TS-GCs by qRT-PCR analysis (Fig. 3G). To investigate the potential regulation of *CTPS2* expression by the apelin/APLNR pathway, we conducted an experiment where control-GCs were treated with APLN-13 from differentiation day 3 to day 14. By qRT-PCR analysis, we observed that the mRNA levels of *APLN*, *CTPS2*, as well as other granulosa cell marker genes such as *FOXL2*, *AMHR2* and *CYP19A1*, were upregulated after APLN-13 treatment (Fig. 3H). However, the expressions of *APLNR* and *FSHR* were downregulated, suggesting the presence of a feedback loop that controls the mRNA expressions of *APLNR* and *FSHR* upon APLN-13 treatment (Fig. 3H). While the *CTPS2*, *FOXL2*, *CYP19A1* were downregulated expressed in the APLN-13 cotreatment with APLNR antagonist (ML221) (Fig. 3H). In conclusion, we suggest that the apelin/APLNR pathway targets *CTPS2* expression potentially to support cell proliferation of granulosa cells.

### Activation of the apelin/APJ pathway can rescue the dysregulation of cell divisions in TS-GCs

**Fig. 4:**
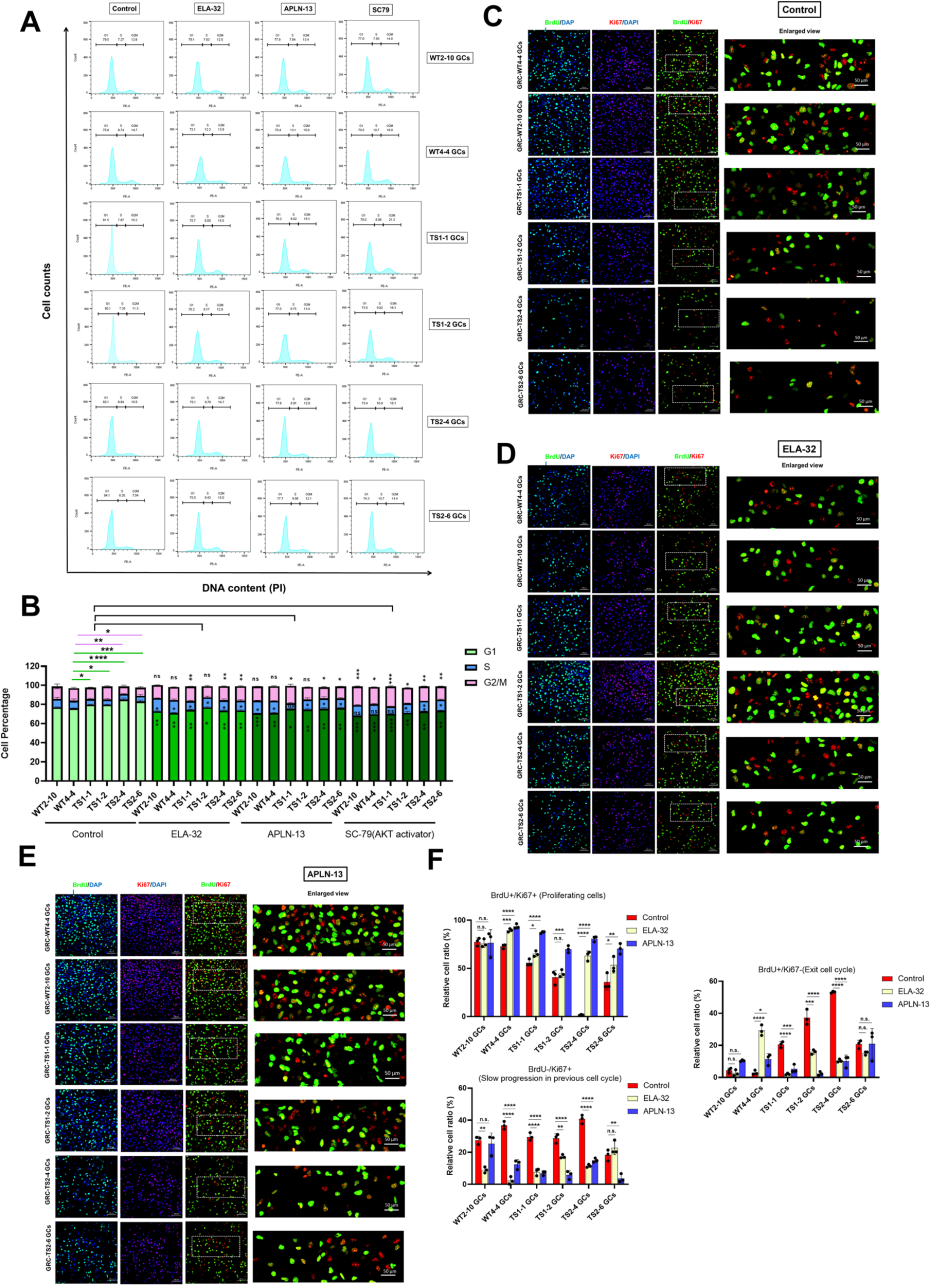
Apelin signaling can partially restore the cell growth and cell division defects in TS-GCs. **A** Cell cycle analysis using PI staining were performed in WT control GCs and TS-GCs. **B** The quantification of cell percentages in each cell cycle in G1, S, and G2/M phases. Error bars indicate the mean ± SD (*n* = 3). One-way ANOVA with a Dunnett post hoc test was performed. n.s. not significant, *p < 0.05, **p < 0.01, ***p < 0.001, and ****p < 0.0001 vs. GRC-WT4-4 GCs. Representative fields of colocalization of anti-BrdU (green color) and anti-Ki67 (red color) staining results in vehicle control (**C**), treatment with ELA-32 (**D**), and treatment with APLN-13 (**E**). **F** Relative cell ratio in percentage rate in each treatment condition were calculated as proportions of the total signal in green and red channel (in BrdU+Ki-67+ panel) or DAPI signal (in BrdU+Ki-67− and in BrdU-Ki-67+ panel). Proliferating cells are cells labeled with both Ki-67 and BrdU, while non-proliferating cells were labeled with only DAPI. Cells exiting the cell cycle were labeled with BrdU but not Ki-67. Error bars indicate the mean ± SD (*n* = 3). One-way ANOVA with a Dunnett post hoc test was performed. Multiple comparisons between treatment vs. control group. n.s. not significant, *p < 0.05, **p < 0.01, ***p < 0.001, and ****p < 0.0001.

Since granulosa cells play a crucial role in supporting folliculogenesis during female germ cell development, it is important to understand whether their cell expansion ability is altered in Turner syndrome-differentiated granulosa cells (TS-GCs). To investigate this, we performed cell cycle analysis using PI staining in GC samples and compared the results with control GCs (Fig. 4A). We found that TS-GCs exhibited a higher percentage of cells in the G0/G1 phase, indicating a larger population of cells in the resting phase. In contrast, the percentage of cells in the S phase, indicating the proliferated cell population, was lower in TS-GCs compared to healthy control granulosa cells (GCs) (Fig. 4B). These findings suggest that TS-GCs may have altered cell cycle dynamics with a decreased proportion of cells actively progressing through the cell cycle and an increased number of cells in a resting state. We found *CTPS2* was potentially the downstream target of apelin receptor (APJ), where APJ has two established specific endogenous ligands, apelin and *ELABELA* (ELA). *ELABELA* was identified to play a crucial role in embryonic development [32, 33]. *ELABELA* expression was also downregulated in TS-GCs (Fig. 3G). We next investigated whether the addition of APJ ligands, ELA-32 and APLN-13, could rescue the phenotype of TS-GCs. We observed that the dysregulation of the cell cycle in TS-GCs could be rescued by the addition of ELA-32 or APLN-13 (Fig. 4A, B). Additionally, to activate the downstream signaling pathway of APJ, namely the AKT pathway, compound SC-79 was used, and we observed partial rescue of the dysregulated cell cycle, particularly in the G1 and G2/M phase (Fig. 4A, B).

We also used combined analysis of BrdU uptake alongside with Ki-67 expression to measure the distinct parameters of cell division. During cell division, BrdU, a thymidine analog, is integrated into the DNA of the dividing cells. As a result, cells that have undergone division during the labeling period are permanently labeled by BrdU. Cell proliferation antigen Ki-67 (also known as Ki67 or Ki-67) is only present in dividing cells. The expression levels and localization of the Ki-67 protein vary throughout the cell cycle, with the highest expression observed during the G2 phase and mitosis [34, 35]. Ki67 is essential for the localization of nucleolar granular components to mitotic chromosomes during cell division and is linked to heterochromatin organization in proliferating cells [36, 37]. GCs were treated with BrdU for 6–7 h and subjected to fixation and analyzed by co-staining with anti-BrdU antibody and anti-Ki67 antibody. Ki67+/BrdU+ cells are indicative of newly divided cells that underwent cell division during the BrdU treatment period. When a cell population is Ki67+/BrdU−, it could be an indication of inadequate uptake of BrdU or the absence of cell division during the BrdU treatment period. In this context, it can be inferred that these cells underwent slow progression in the preceding round of the cell cycle. Ki67−/BrdU+ cells suggest that cells underwent division at some point during the BrdU treatment period, but the absence of Ki67 expression implies that this cell division is at a standstill. We found that there was a higher Ki67−/BrdU+ and Ki67+/BrdU− percentage of cells in TS-GCs as compared with control-GCs (Fig. 4C). In contrast, a lower ratio of BrdU+/Ki67+ cells in the TS-GC samples was observed compared to healthy control GCs (Fig. 4C). However, the Ki67+/BrdU+ ratio in TS-GCs was increased after supplementation of ELA-32 (Fig. 4D, F) or APLN-13 (Fig. 4E, F). These data indicate that the apelin pathway can partially rescue the cell division defect of TS-GCs.

### Apelin/APJ pathway activation partially ameliorates the downregulation of GC marker genes and restores *CTPS2* expression

**Fig. 5:**
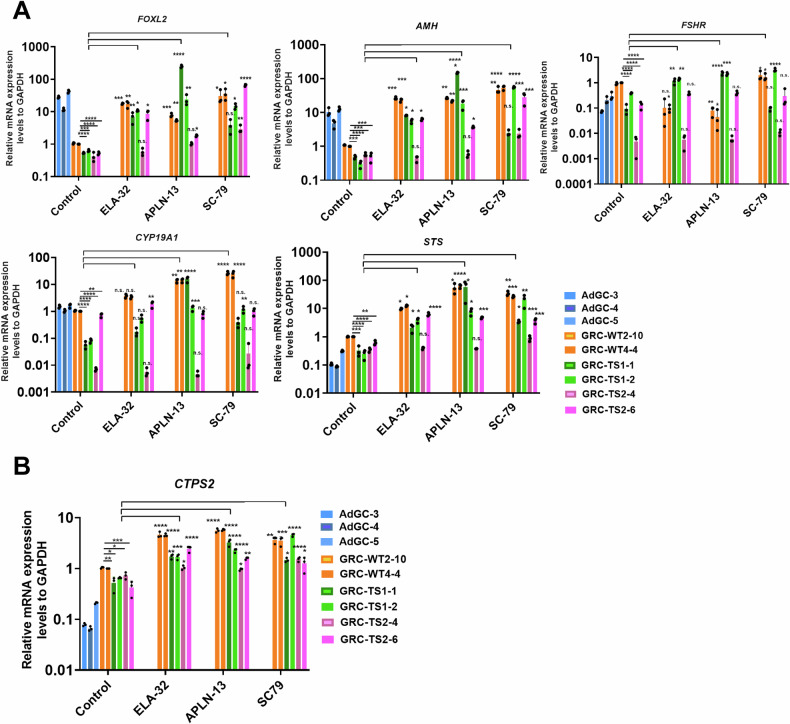
Activation of apelin pathway ameliorates the expression of GC markers along with CTPS2 restoration. **A** qRT-PCR analysis of GC marker gene expression at differentiation day 14. **B** qRT-PCR analysis GCs at differentiation day 14 regarding the levels of the CTPS2 gene after ELA-32, APLN-13, or SC79 treatments. Error bars indicate the mean ± SD (*n* = 3). One-way ANOVA with a Dunnett post hoc test was performed. Multiple comparisons between treatment vs. control group. n.s. not significant, *p < 0.05, **p < 0.01, ***p < 0.001, and ****p < 0.0001.

Beyond cell division and proliferation, we investigated whether apelin/APJ pathway activation could influence the downregulation of granulosa cell-expressed genes. ELA-32, APLN-13, and SC79 were supplied into the culture medium from differentiation day 4 to day 14. Interestingly, APLN-13 and SC79 exhibited notably superior effects in rescuing the expression of granulosa cell marker genes such as *FOXL2*, *AMH*, *CYP19A1*, and *STS* (steroid sulfatase), which is a metabolic enzyme that synthesizes 3-beta-hydroxysteroid sulfates, which serve as metabolic precursors for estrogens, androgens, and cholesterol (Fig. 5A). Furthermore, *CTPS2* gene expression was fully restored following the supplementation of ELA-32, APLN-13, and SC79 in TS-GCs (Fig. 5B). This outcome suggests that CTPS2 is a downstream target of the apelin receptor (APJ).

## Discussion

In vitro modeling of reproductive lineage cells using iPSCs is an attractive methodology to understand the crucial factors important for cell differentiation in embryogenesis. In this study, we demonstrated the successful derivation of iPSCs from fibroblasts with Turner syndrome and then tried to optimize the differentiation protocol of the granulosa cells to up to around 80% compared to previous reports which only achieved 4–20% [21, 38, 39] (growth factor-based). In transcriptional factor-based induced differentiation, 8–50% in 6 cell lines or 79% in one cell line depending on different clones [40], but very low FSHR expression was detected in these cells. We found that iPSC lines reprogrammed from TS patients showed core pluripotency marker expression, such as *OCT4*, *SOX2*, *NANOG* and have the capability to differentiation into three germ lineage cells using spontaneous embryonic bodies (EBs). However, we found that the granulosa cell differentiation abilities were lower in TS-iPSCs compared to WT-iPSCs. During the embryonic period, a decrease in cell division activity and lower cell numbers could affect cell differentiation and the process of organogenesis. Cell cycle dysregulation was also observed in the cells displaying an increased number of cells in a G1 phase and decreased percentages of cells with BrdU+/Ki67+ staining (Fig. 4). We suggest that at least for the 45,XO karyotype that the pre-granulosa cells in the embryonic gonads may be proliferating slowly and have poor differentiation potential.

In our bulk transcriptome analysis, the differentiated cells were not isolated based on surface markers before being subjected to the transcriptomic experiment. Therefore, we cannot directly attribute the differences in differentiation abilities between control iPSC lines and Turner syndrome iPSC lines solely to a cell-autonomous defective pathway or an overall downregulated differentiation ability. It is challenging to determine which factor is the cause and which is the effect, as both could contribute to the disease phenotype.

The hypothesis that X chromosome-linked gene haploinsufficiency, e.g., where a single normal allele does not provide enough function, is linked to Turner syndrome (TS) phenotypes is an area of active research. In this study, we found that *APLN*, *ELABELA* and *CTPS2* are significantly downregulated in granulosa cells differentiated from TS-iPSCs compared to wild type control cells (Fig. 3G). The *APLN* gene, which is located in Xq26.1 region, encodes the precursor apelin proteins. *APLN* is subjected to X-chromosome inactivation (XCI) [41]. *ELABELA* is encoded by the *ELBELA* gene at 4q32.3 and is important for the embryonic cardiovascular system and early placental development [33, 42]. The *CTPS2* (CTP Synthase 2) gene encodes a protein catalyzing the formation of CTP from UTP, which is the precursor necessary for the synthesis of RNA and DNA. *CTPS2* resides in the Xp22.2 region and is an X-chromosome inactivation (XCI) escapee conserved across species with no known Y homology [43]. In the GESA analysis based on the Molecular Signatures Database (MSigDB) dataset for positional gene set (C1) information, the most significantly downregulated and highest numbers of genes are in the chromosomal Xp22, Xq26, Xq22, Xq23, Xq13 regions, which implies that these differentially expressed genes are dosage sensitive genes contributing to the TS phenotype once they lose the second X copy. Although our observations may be informative, how XCI affects the imbalance of gene dosage and correlates with genes important for reproductive phenotypes in TS patients is not the main focus of our current study, where we have chosen to specifically focus on the newly discovered role of apelin/APJ as contributing TS phenotypes.

The role of apelin/APJ in the regulation of cardiovascular function [44, 45], in vasculogenesis, increased expressions in human [46], porcine [47], bovine [48] ovaries during FSH or LH treatments, and placental development and pregnancy [32, 49] have all been reported. There is evidence indicating widespread expression of apelin and its receptors in the hypothalamic nuclei (H), particularly the paraventricular nucleus (PVN) and supraoptic nucleus (SON), which are associated with the release of gonadotropin-releasing hormone (GnRH) [50]. Recombinant human APLN-13 and APLN-17 have both been reported to increase progesterone and estradiol secretion in a dose-dependent manner [46]. These studies imply that both ovary tissues and the HPG axis are important targets of apelin/APJ signaling. In our study, we observed that reactivation of the apelin pathway through APLN-13 or ELA-32 supplements can rescue the cell-cycle dysregulation and GC marker gene expression in TS-differentiated granulosa cells (TS-GCs) (Fig. 4). Therefore, a protractive cell cycle in GCs could signify the initiation of the pathogenesis of Turner syndrome, triggering a cascade of phenotypic consequences that may manifest as early as the embryonic/fetal development stage in females.

iPSC clones, even when originating from the same individual, are anticipated to exhibit genetic heterogeneity. Indeed, the Human Induced Pluripotent Stem Cells Initiative (HipSci) has found that a significant portion, approximately 5-46%, of the variability in iPSC cell characteristics is primarily attributed to variances between different individuals [51]. The observation of differing degrees of phenotypic performance and slight variations in response to ligand treatments between TS1 and TS2 samples might explain individual distinctions based on their unique genetic backgrounds which leads to limited restored effects in a subclone (Fig. 5). Nevertheless, our findings suggest that the apelin/APJ pathway is defective in embryonic granulosa cells of TS patients, as revealed using an iPSC model. Additionally, we have developed and enhanced a granulosa differentiation protocol, offering potential benefits for future reproductive studies and medical applications.

## Materials and methods

### Patient recruitment and ethical approval

Primary human granulosa cells (GCs), commonly referred to as cumulus cells, were procured from the follicular fluid contained within the oocyte-granulosa cell complex. These cells were sourced from two distinct groups: (1) GCs were collected from women who were part of infertile couples seeking IVF treatment, and (2) GCs were also obtained from healthy women who were undergoing oocyte cryopreservation procedures. The study encompassed women aged between 29 and 45 years who met the criteria, and their participation was secured with informed consent obtained from the patients at the Taipei Infertility Center. Nonetheless, it is important to note that certain conditions led to the exclusion of participants from the study. Women who had received a diagnosis of unexplained premature ovarian failure or were presently experiencing amenorrhea were not eligible for inclusion in this study. This study was reviewed and approved by the Institutional Review Board (IRB) at Academia Sinica (AS-IRB01-22054). We also confirm that all methods were performed in accordance with the relevant guidelines and regulations.

### Cellular reprogramming and human iPSCs (hiPSCs) culture

Fibroblasts from two unrelated, unaffected individuals and two individuals with Turner syndrome (TS) were purchased from the Coriell Institute for Medical Research and cultured in MEM (Minimum Essential Medium) with 15% fetal bovine serum (FBS) (Thermo Fisher Scientific, 11095080). Cellular reprogramming was performed using the iPSCs CytoTune™-iPS 2.0 Sendai Reprogramming Kit, following the manufacturer’s instructions (Thermo Fisher Scientific, A16517). The generated hiPSC lines corresponding to each original fibroblast source are listed as follows: GRC-WT2-10 (AG02102); GRC-WT4-4 (AG08470); GRC-TS1-1 (GM01176); GRC-TS1-2 (GM01176); GRC-TS2-4 (GM06563); GRC-TS2-6 (GM06563). After viral transduction, fibroblasts were seeded onto mitomycin-c treated mouse embryonic fibroblasts (MEFs) cultured on 0.2% gelatin-coated plates. Approximately 20–25 days after viral transduction, each single iPSC colony was manually picked and maintained on inactivated MEFs in a 37 °C incubator with 5% CO_2_ in a humidified atmosphere. The hiPSC culture medium consisted of DMEM/F12 (Thermo Fisher Scientific, 11330032) supplemented with 20% KnockOut serum replacement (Gibco, 10828028), 10 ng/mL bFGF (Gibco, 100-18B-1MG), 1× MEM Non-Essential Amino Acids Solution (Gibco, 11140035), 1× GlutaMAX™ Supplement (Gibco, 35050061), and 0.1 mM β-mercaptoethanol (Sigma, M3184). The medium was changed daily. Cells were passaged every 5–7 days with 0.1% collagenase, type IV (Thermo Fisher Scientific, 17104019) at 37 °C for approximately 30 min to lift the hiPSC colonies. Colonies were suspended and washed with hiPSC culture medium and then split at a ratio of 1:3–1:6 onto inactivated MEFs.

### Granulosa cell differentiation from hiPSCs

After hiPSC colonies had grown on MEFs for approximately 5–7 days, the colonies were incubated and lifted with 0.1% collagenase, type IV (Thermo Fisher Scientific, 17104019), at 37 °C for about 30 min. Subsequently, the colonies were suspended, and any remaining collagenase was washed out with hiPSC culture media. In this study, 4 conditions were tested as shown in Fig. 1C. On day 0, cell colonies were re-suspended in embryonic body (EB) basal medium, consisting of DMEM/F12 (Thermo Fisher Scientific, 11330032) supplemented with 20% KnockOut serum replacement (Gibco, 10828028), 4 ng/mL bFGF (Gibco, 100-18B), 1× MEM Non-Essential Amino Acids Solution (Gibco, 11140035), 1× GlutaMAX™ Supplement (Gibco, 35050061), and 0.1 mM β-mercaptoethanol (Sigma, M3184). Additionally, 10 μM Y-272632 (LC Laboratories, Y-5301) was added to the culture medium. On day 2, the medium was replaced with EB basal medium supplemented with small molecules and proteins based on each condition: condition #1: 10 ng/mL BMP4 (BioLegend, 795606); condition #2: 2.5 ng/mL BMP4 and 10 μM SB4 (MedChemExpress, HY-124697); condition #3 and condition #4: 3 μM CHIR99021(Lc laboratories, C-6556). From day 3 to day 6, the medium was replaced with EB basal medium supplemented with small molecules and proteins based on each condition: condition #1: 10 ng/mL BMP4, 6 ng/mL Human WNT3A (Prospec, CYT-861), 6 ng/mL activin A (BioLegend, 796206), 5 ng/mL bFGF (Gibco, 100-18B); condition #2: 5 ng/mL BMP4, 10 μM SB4, 6 ng/mL Human WNT3A, 6 ng/mL activin A, 5 ng/mL bFGF, and 3 μM CHIR99021; condition #3 and condition #4: 3 μM CHIR9902. Meanwhile, the culture plates were put onto a horizontal shaker at 80 rpm. This medium was refreshed every 48 h until day 7, at which point the medium was replaced with maturation medium containing EB basal medium supplemented with small molecules and proteins based on each condition: condition #1: 10 ng/mL BMP4, 5 ng/mL bFGF, and follistatin 288 (R&D, 5836-FS-0251/CF); condition #2 and condition #4: 5 ng/mL BMP4, 10 μM SB4, 5 ng/mL bFGF, and follistatin 288; condition #3: 10 ng/mL BMP4, 1 μg/mL heparin (Sigma-Aldrich, H3149), 50 ng/mL GDF9 (R&D Systems, 8266-G9-010/CF), and 25 ng/mL BMP15 (R&D Systems, 5096-BM-005). This medium was changed every 48 h. On day 10, approximately 10–20 GC-like spheroids were replated into one well of a 0.2% gelatin-coated 6-well plate for another 4 days. For the apelin ligand treatment experiment, 5 μM ELA-32 (MedChemExpress, HY-P2196A), 10 μM [Tyr0]-Apelin-13 (aapptec, P000849), and 10 μM SC79 (MedChemExpress, HY-18749) were added separately into the culture medium from day 3 to day 14.

### RNA extraction and qRT-PCR assays

Cells were lysed by 1 mL of TRIzol™ Reagent (Thermo Fisher Scientific, 15596018). 0.2 mL of chloroform was added per 1 mL of TRIzol™ Reagent to promote phase separation and to separate RNA from proteins and lipids. Cells were vortexed vigorously using a vortex mixer for 15 s. Sample(s) were incubated for 15 min on ice and centrifuged for 30 min at 12,000 × *g* at 4 °C to separate RNA from the rest of the tissue/cell lysate. The upper aqueous phase was carefully removed and transferred to a fresh tube. The volume of the upper aqueous phase was recorded, and an equal amount of isopropanol was added to the aqueous solution. The samples were centrifuged for 30 min at 12,000 × *g* at 4 °C. The supernatant was removed without touching the RNA pellet at the bottom of the tube. The RNA pellet was washed with 1 mL of 75% ethanol and was prepared with nuclease-free water (Thermo Fisher Scientific, AM9932) and centrifuged for 15 min at 12,000 × *g* at 4 °C. Finally, the sample was washed again with 1 mL of absolute ethanol by centrifuging for 15 min at 12,000 × *g* at 4 °C. The RNA pellet was air-dried at room temperature for at least 30 min. The RNA was dissolved in nuclease-free water. For relative gene expression analysis by qRT-PCR, genomic DNA removal and cDNA synthesis were conducted using the TOOLSQuant II Fast RT Kit (Biotools, KRT-BA06). An equivalent amount of cDNA to 50 ng of RNA was used per real-time PCR reaction mixed with KAPA SYBR FAST qPCR Master Mix (2X) ROX Low (KAPA Biosystems, KK4619). The 2^(-DD C(T)) method was employed, with glyceraldehyde-3-phosphate dehydrogenase (GAPDH) as a reference gene, to calculate relative gene expression levels between different samples. The primer sequences were listed in Table 1.

**Table 1:** qRT-PCR primer.

Gene name	Forward primer sequence (5’->3’)	Reversed primer sequence (5’->3’)
*GATA4*	GGTCACTATCTGTGCAACGC	GTTTGGATCCCCTCTTTCCG
*LHX1*	AGAACGACTTCTTCCGGTGT	GGTGAAACACTTTGCTCCGC
*OSR1*	CGGTGCCTATCCACCCTTC	GCAACGCGCTGAAACCATA
*PAX2*	CAAAGTTCAGCAGCCTTTCC	CCACACCACTCTGGGAATCT
*FOXL2*	GGTCGCACAGTCAAGGAGC	CGCGATGATGTACTGGTAGATG
*AMHR2*	CGACCACATTGTCCGATTTATCA	CCCTTGGGATGCAGTTCCA
*CYP19A1*	TGCATGGGAATTGGACCCC	GGTTGTAGTAGTTGCAGGCAC
*FSHR*	TCTGGCAGAAGACAATGAGTCC	TGAGGATGTTGTACCCCATGATA
*AMH*	CGCTGCTTCACACGGATGACC	GGTGGCGACTCCTCGAGTTCC
*FSH*	ACACCACTTGGTGTGCTGGCTA	CAAGGAATCTGCATGGTGAGCAC
*CYP11A1*	TGGCATCCTCTACAGACTCCTG	CTTCAGGTTGCGTGCCATCTCA
*STS*	TCCCGCACTGGAGTTTTCC	CCGTGATGTAAAGGGTGGTGA
*APLN*	GTCTCCTCCATAGATTGGTCTGC	GGAATCATCCAAACTACAGCCAG
*APLNR*	CCTGCATCAGCTACGTCAACA	GGGATGGATTTCTCGTGCATCT
*ELABELA*	CACGAGTACCCTTTCCCTGA	GGCTGGGTGTCTTTCCTTC
*CTPS2*	CGATGCTGGCACTTTTTCACC	ATGTGAGGGACAACTTGCACT
*GAPDH*	CATCACCATCTTCCAGGAGC	ATGCCAGTGAGCTTCCCGTTC

### Flow cytometry analyses

Cells were detached using 0.05% trypsin and a uniform single-cell suspension was obtained by passing cell suspensions through a 40 μm cell strainer (Falcon, 352340). They were then fixed with a final concentration of 4% paraformaldehyde on ice for 10 min. After washing the cells twice with 1x PBS, aliquots of up to 1 × 10^6^ cells per reaction were placed into FACS tubes (5 mL round-bottom polystyrene tubes). The cells were suspended in 100 µL of permeabilization buffer (0.3% saponin/1x PBS) on ice for 30 min to enable intracellular staining without altering membrane antigen expression. After this incubation, the cells were washed once with 1x PBS and centrifuged at 2000 rpm for 5 min. Single cells were first incubated with primary antibodies (listed in Table 2) at the appropriate dilution in 3% FBS/1x PBS at 4 °C overnight. Cells were then washed twice with 1 mL of washing buffer (0.1% saponin/1% BSA/1x PBS) and centrifuged at 2000 rpm for 5 min. Subsequently, the cells were incubated for 2 h at room temperature in the dark with fluorochrome-conjugated antibodies (listed in Table 2) diluted in 1% BSA/1x PBS, followed by two more washes with the washing buffer. Finally, the cells were suspended in 1 mL of 1x PBS and made ready for analysis. Isotype IgGs were used as negative controls. All specimens were analyzed using a BD FACSCanto II flow cytometer (Becton Dickinson).

**Table 2:** Antibodies used in this study.

Antibodies	Brand	Catalog number	Purpose	Working concentration
GATA4 antibody	Santa Cruz	sc-25310	IF	1:100
AMHR2 antibody	Thermo Fisher Scientific	PA5-112901	IF, flow	1:100, 1:50
FSHR antibody	Proteintech	22665-1-AP	IF, flow	1:50, 1:50
FOXL2 antibody	Abcam	ab246511	IF	1:100
PAX2 antibody (clone: Poly19010)	BioLegend	901001	IF	1:100
CYP19A1 antibody	Santa Cruz	sc-374176	IF, flow	1:100, 1:50
BrdU antibody (clone: BU-33)	Sigma	B2531	IF	1:200
Ki-67 antibody (clone: SP6)	Thermo Fisher Scientific	MA5-14520	IF	1:200

### Cell cycle analysis using propidium iodide (PI) staining

Cells were harvested and washed in 1x PBS. Cells were fixed in −20 °C 70% ethanol prepared using distilled water to prevent protein precipitation during fixation. Aliquots of up to 1 × 10^6^ cells/100 μL were added into FACS tubes. The 1 mL of cold 70% ethanol was added dropwise to the cell suspension (100 μl) while gently vertexing. Fixation was performed for 30 min at 4 °C. The cells were washed twice with 1x PBS and centrifuged at 850 × *g* at 4 °C, taking care to avoid cell loss when discarding the supernatant, especially after spinning out the ethanol. A total of 200 µL of PI Staining Solution (Sigma, P4170) (made from a 50 µg/mL PI stock solution in 1x PBS) was added and 50 µL of a 100 µg/mL stock of RNase A was added per sample. Samples were incubated at 4 °C overnight and protect them from light. All specimens were analyzed using a BD FACSCanto II flow cytometer (Becton Dickinson). Each cell cycle phase was analyzed by the PI histogram plot.

### Immunofluorescence staining

The EB spheroids or GC-like cells were fixed in a 4% paraformaldehyde (PFA) solution for 30 min at room temperature (RT). After three washes with 1× PBS to remove the PFA, these cells were further incubated in a 10% donkey serum solution with 0.5% Triton X-100 in 1× PBS for 30 min at RT. This was followed by incubation with the respective antibodies (Table 2) in a 5% donkey serum staining solution with 0.1% Triton X-100 in 1× PBS overnight at 4 °C. Subsequently, the cells were washed with 1× PBS twice, and secondary antibodies were applied for 2 h at RT. The nuclei were stained with DAPI at a final concentration of 0.5 μg/mL for 10 min. After that, the cells were mounted using ProLong™ Diamond Antifade Mountant with DAPI (Thermo Fisher Scientific, P36962). The antibodies used are listed in Table 2. The LSM880 confocal imaging system was used to capture and visualize the results.

### BrdU labeling and Ki-67 staining

BrdU was added to the GC culture medium on day 14 at a final concentration of 10 µM and incubated at 37 °C for 5–6 h. The medium was aspirated, and cells were fixed for 10 min with 4% paraformaldehyde (PFA) at RT. After washing the cells with 1x PBS three times, 2 M HCl (prepared by diluting 1:5 from a 12 M HCl stock solution in distilled water) was added to each well for a further 30-min incubation at RT. This treatment denatures double-stranded DNA into single strands, allowing the primary antibody to penetrate the incorporated BrdU. Subsequently, cells were washed three times for 10 min each with 1x PBS, followed by blocking nonspecific epitopes through incubation in blocking buffer (10% donkey serum solution with 0.5% Triton X-100 in 1× PBS) for 30 min at RT. Anti-BrdU and anti-Ki-67 antibodies (see Table 2), diluted in staining buffer, were added overnight with gentle shaking at 4 °C. The next day, cells were washed three times with 1x PBS and incubated at RT for 2 h with secondary antibodies and DAPI diluted in staining buffer. Finally, cells were mounted using ProLong™ Diamond Antifade Mountant with DAPI. The colocalization of BrdU and Ki-67 was calculated as a relative ratio normalized with the DAPI signal using Image J software.

### Anti-Müllerian hormone (AMH) ELISA assay

Briefly, GC culture supernatant was collected on differentiation day 14 and centrifuged at 2500 rpm at 2–8 °C for 5 min to remove cell debris. The clarified cell culture supernatants were aliquoted and stored at −80 °C for future analysis. The human AMH (Müllerian-inhibiting factor) ELISA Kit (FineTest, EH0528) was used according to the manufacturer’s instructions.

### Bulk-RNA sequencing analysis

RNA-sequencing (RNA-Seq) analysis was performed on differentiation day 14 GCs and cumulus cells. Total RNA was extracted using the DNA-Free RNA Kit (Zymo Research) following the manufacturer’s instructions. Stranded mRNA-Seq libraries were prepared using the Illumina TruSeq Stranded mRNA Library Prep Kit, as per the manufacturer’s instructions. Sequenced reads were quality-tested using FASTQC. Kallisto [52] was used for the pseudoalignment algorithm to determine the abundance of transcripts in a sample. The final output of transcripts per million (TPM) and estimated counts was obtained. Transcript abundance files were used as input for differential expression gene (DEG) analysis, performed by the DESeq2 package [53] version 1.43.0. Subclones of GCs differentiated from individual iPSCs were set as biological replicates. DEGs were defined as having a false discovery rate (FDR) < 0.05 and a log_2_ fold change >1. The raw data was deposited in GEO: GSE271780.

### GSEA analysis

WebGestalt [54] was used to analyze the gene set analysis by web-based tools (https://www.webgestalt.org/#).

## Supplementary information

Supplement figures

## Data Availability

All data supporting the findings of this study are available from the corresponding author on reasonable request.
