# Supplementary material for: Identification of apelin/APJ signaling dysregulation in a human iPSC-derived granulosa cell model of Turner syndrome
Source: Cell Death Discov. 2024 Nov 14;10:468. doi: 10.1038/s41420-024-02231-9 (PMC11564969; doi:10.1038/s41420-024-02231-9)
Supplement: Supplementary file 1 — Supplement figures [file 41420_2024_2231_MOESM1_ESM.pdf]

Supplement figures

Identification of apelin/APJ signaling dysregulation in a human iPSC-derived granulosa cell model of Turner syndrome

Wei-Ju Chen[1], Yi-Ya Chao[1], Wei-Kai Huang[1], Wei-Fang Chang[2,3], Chii-Ruey Tzeng[2,3,4], Chi-Hsuan Chuang[1], Pei-Lun Lai[1], Scott C. Schuyler[5], Long-Yuan Li[6,10], Jean Lu[1,7,8,9,10]

Affiliations

[1] Genomics Research Center, Academia Sinica, Taipei 11529, Taiwan

[2] Taipei Fertility Center, Taipei 110, Taiwan

[3] Taipei Medical Technology Co., Ltd., Taipei 110, Taiwan

[4] Department of Obstetrics and Gynecology, School of Medicine, College of Medicine, Taipei Medical University, Taipei 110301, Taiwan

[5] Department of Biomedical Sciences, College of Medicine, Chang Gung University, Division of Head and Neck Surgery, Department of Otolaryngology, Chang Gung Memorial Hospital, Taoyuan 33302, Taiwan

[6] Department of Life Sciences, National Chung Hsing University, Taichung 402202, Taiwan

[7] Department of Biomedical Sciences and Engineering Tzu Chi University, Hualien 97004, Taiwan

[8] Graduate Institute of Medical Sciences, National Defense Medical Center Taipei 11490, Taiwan

[9]Genomics and System Biology Program, College of Life Science, National Taiwan University,10617, Taiwan

[10] Corresponding author: Jean Lu, Long-Yuan Li

Phone: +886-2-2787-1247

Email: jeanlu@gate.sinica.edu.tw; lyuan@dragon.nchu.edu.tw

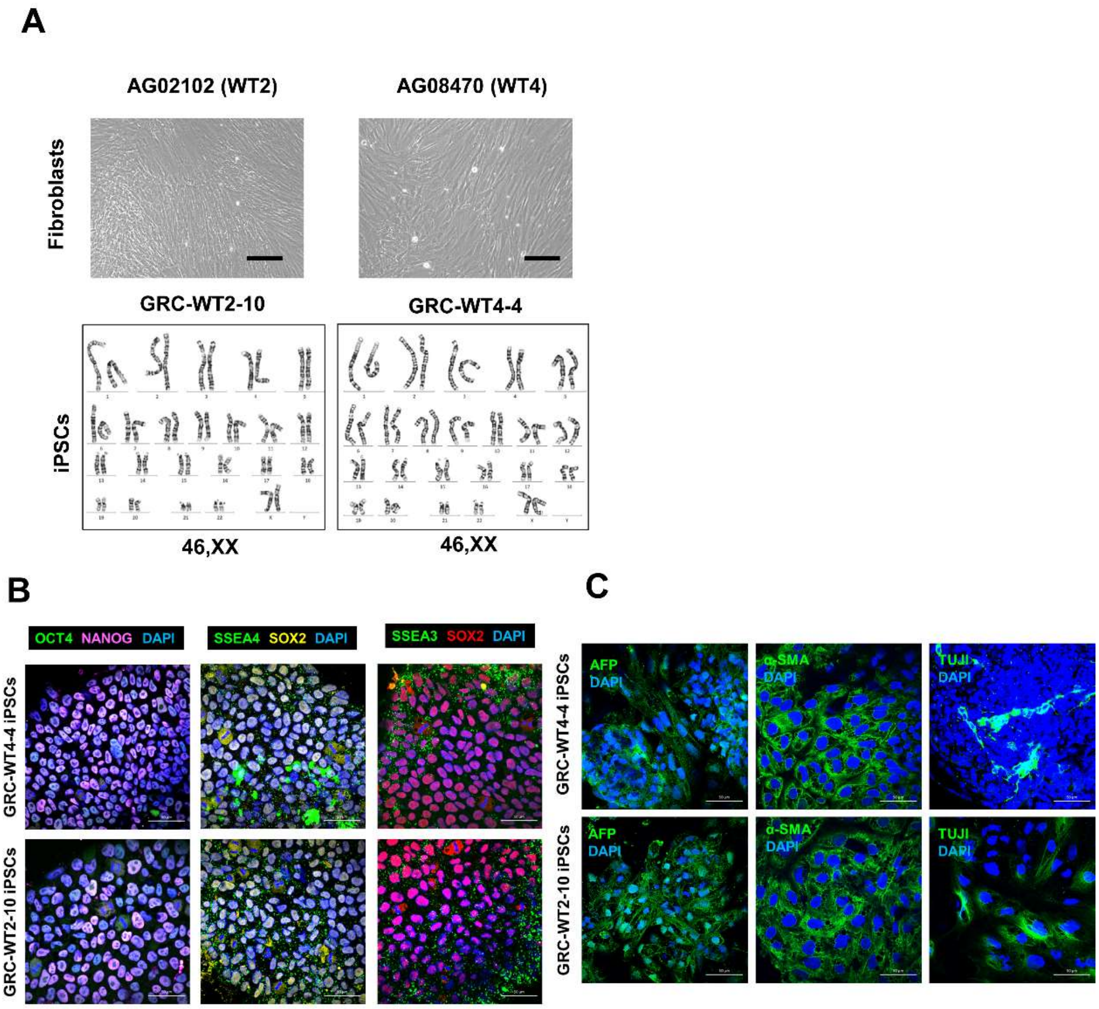

Fig. S1 Characterization of iPSC reprogramming from healthy donor fibroblasts.

(A) Karyotype analyses revealed that normal 46,XX in GRC-WT2-10 iPSCs and GRC-WT4-4 iPSCs reprogramming from parental fibroblasts. **(B)** Immunofluorescence staining for pluripotency marker, OCT4, SOX2, NANOG, SSEA4, SSEA3. Scale bar, 50 μm. **(C)** An *in vitro* spontaneous differentiation assay using embryonic bodies (EBs) formation (10% FBS, 10 days) was performed and immunofluorescence staining for the trilineage markers for ectoderm (TUJ1),

endoderm (AFP), and mesoderm (α-SMA). Scale bar, 50 μm.
